# Regulation of eukaryotic gene expression by the untranslated gene regions and other non-coding elements

**DOI:** 10.1007/s00018-012-0990-9

**Published:** 2012-04-27

**Authors:** Lucy W. Barrett, Sue Fletcher, Steve D. Wilton

**Affiliations:** Centre for Neuromuscular and Neurological Disorders (CNND), The University of Western Australia (M518), 35 Stirling Highway, Crawley, WA 6009 Australia

**Keywords:** Regulation, Expression, Non-coding, Untranslated, RNA, Control

## Abstract

There is now compelling evidence that the complexity of higher organisms correlates with the relative amount of non-coding RNA rather than the number of protein-coding genes. Previously dismissed as “junk DNA”, it is the non-coding regions of the genome that are responsible for regulation, facilitating complex temporal and spatial gene expression through the combinatorial effect of numerous mechanisms and interactions working together to fine-tune gene expression. The major regions involved in regulation of a particular gene are the 5′ and 3′ untranslated regions and introns. In addition, pervasive transcription of complex genomes produces a variety of non-coding transcripts that interact with these regions and contribute to regulation. This review discusses recent insights into the regulatory roles of the untranslated gene regions and non-coding RNAs in the control of complex gene expression, as well as the implications of this in terms of organism complexity and evolution.

## Introduction

**Fig. 1:**
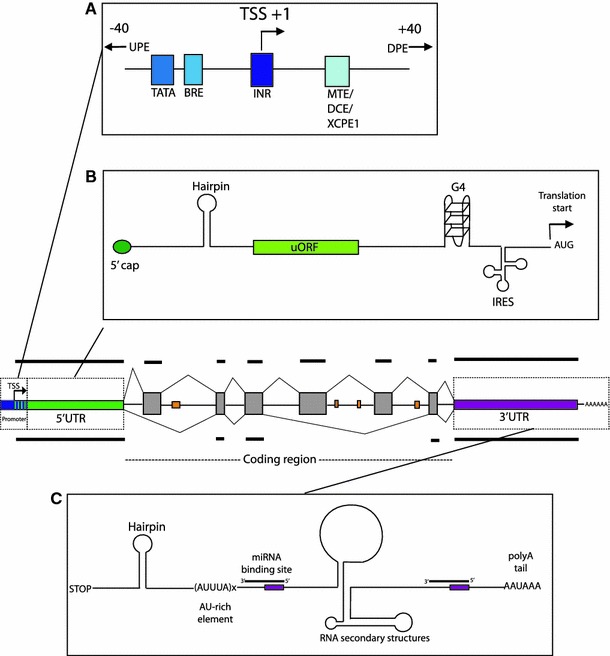
Regulatory elements within the noncoding gene regions. The *centre image* shows a typical gene, with exons indicated in *grey*. The *orange rectangles* indicate intronic enhancer elements. **a** Promoter region regulatory elements (adapted from [162]). Upstream and downstream promoter elements situated outside of the core promoter region are indicated by the *arrows*. **b** Regulatory elements in the 5′UTR. **c** Regulatory elements in the 3′UTR

Over the last decade, it has become increasingly apparent that regulation of gene expression in higher eukaryotes is a complex and tightly regulated process involving many different factors and levels of control. For a given gene, the untranslated gene regions, including the 5′ and 3′ untranslated regions (UTRs), and introns are the major regions involved in the regulation of expression (Fig. 1). Despite being dismissed as “junk” DNA for many years, intergenic regions have also been found to contribute to control of gene expression, and evidence of pervasive transcription throughout the genome [14, 19, 30], both sense and antisense [71], implicates a role for all regions of the genome. Accumulated evidence indicates that the complexity of higher organisms, which correlates with an increase in the size of non-coding regions, arises from an increase in the number and complexity of regulatory pathways [95], and that it is variation within these non-coding sequences that produces phenotypic variation between both individuals and species [104]. This review will collate current knowledge concerning the role of untranslated gene regions, non-coding RNAs, and other non-coding elements in the control of complex gene expression, with the aim of emphasising the complex mechanisms and interactions involved in precise gene control.

## Promoter

The eukaryotic promoter is a regulatory region of DNA located upstream of a gene that binds transcription factor II D (TFIID) and allows the subsequent coordination of components of the transcription initiation complex, facilitating recruitment of RNA polymerase II and initiation of transcription [79, 162]. The core promoter generally spans ~80 bp around the transcription start site (TSS), and, in mammals, can be separated into two distinct classes: conserved TATA-box enriched promoters that initiate at a single TSS, and variable CpG-rich promoters containing multiple TSS [20]. The latter class is enriched in vertebrates, and expression from these promoters involves the combinatorial effects from a multitude of binding motifs within the promoter region. Some of the major elements involved in regulation by these complex promoters are enhancers, including upstream and downstream promoter elements (UPE and DPEs) that contain transcription factor binding sites, and may act independently or synergistically with the core promoter to facilitate transcription initiation. Also commonly found in complex promoters are B-recognition elements (BRE), which are TFIID recognition elements that aid RNA polymerase II binding, and initiator elements (INR), motifs that can act independently of, and synergistically with, TATA-box promoters via binding of TFIID (for a comprehensive review and details of each element, refer to [79, 162]. Other elements include insulators, activators, repressors, and some rarer, more recently discovered elements such as the motif ten element (MTE), downstream core element (DCE), and the X-core promoter element 1 (XCPE1), all of which act selectively with other elements to contribute to promoter activity (Fig. 1a) [79]. In addition to core elements within the ~80-bp promoter region, identification of general functional regions using deletion analyses in multiple genes implicated the sequence lying −300 to −50 bp of the TSS as generally having a positive effect on promoter activity, while elements that negatively affected promoter activity were located −1,000 to −500 bp upstream of the TSS for 55 % of the genes tested [34].

Genes with complex promoters are likely to make use of regulatory elements, such as enhancers and silencers, selectively, allowing varying levels of expression as required. The *IFN*-*beta* enhancer element has been demonstrated to “loop out” the intervening DNA to access the promoter [131]. This allows specific control of gene activation using general factors. The conformation of the TFIID complex also appears to differ when it is bound to different core promoters, allowing interaction with a large range of subsets of transcriptional activators [162]. A recent study of non-prototypical core promoter recognition factors identified a number of cell-type-specific factors that act in potentiating developmental gene regulation and cellular differentiation [66]. In addition, promoter-selective homologues of basal transcription factors and considerable diversity in the sequence structure and composition of core promoter elements allow complex programs of tissue-specific and promoter-selective transcription, potentially producing a number of specifically expressed gene isoforms [35]. These studies show that promoters in higher organisms are complex regulatory regions consisting of multiple binding elements that can recruit a variety of *cis*-acting regulatory factors as required by the cell.

Promoter usage can have a major impact on gene expression, and many mammalian genes contain multiple promoters [34]. Alternative promoter use is a widespread phenomenon in humans [34] that can alter expression of the associated gene at both the mRNA and protein level. It is also an important mechanism involved in the cell-specific or developmental-specific expression of many genes [95]. For example, TATA-box-lacking and TATA-box-containing alternative promoters of the hemoglobin γ A gene (*HBG1*) are used during and after embryonic development, respectively [44], showing that the basal transcription apparatus can be recruited to different types of core promoters in a developmental stage-specific manner [35]. Another more recent example demonstrates the complexity and variation that can arise through the use of alternative promoters for regulation of the *MITF* transcription factor during vertebrate eye development. Each of the nine alternative promoters associated with expression of this gene produce isoforms containing different first exons and protein binding sites, allowing variable spatial and temporal expression of different protein isoforms during the complex process of eye development [12]. A recent global analysis of mammalian promoters concluded that alternative promoters are over-represented among genes involved in transcriptional regulation and development, while single-promoter genes are active in a broad range of tissues and are more likely to be involved in general cellular processes, such as RNA processing, DNA repair, and protein biosynthesis [7].

Alternative promoter usage has been implicated in the production of biologically distinct protein isoforms [35]. Lymphoid enhancer factor (*LEF1*) is transcribed from two alternative promoters: promoter 1 produces a full length isoform that activates target genes *Wnt/β*-*catenin*, while promoter 2, situated in the intron, produces a shorter isoform that represses target genes [5]. The use of alternative promoters will also affect the 5′UTR, which can alter the stability or translation efficiency of the mRNA variants while encoding identical proteins. *SHOX* (short stature homeobox), a cell-type specific transcription factor involved in cell cycle and growth regulation, uses two alternative promoters producing two distinct 5′UTRs (one is longer and highly structured), resulting in identical proteins that are regulated differently by a combination of transcriptional and translational control mechanisms [15]. These examples confirm that alternative promoter use can play a major role in the spatial and temporal control of gene expression, and that use of alternative promoters is an effective way of increasing the complexity of gene expression pathways.

How promoter selection is determined is not fully understood, but possible mechanisms of promoter switching include diverse core-promoter structure at alternative promoters, variable concentration of *cis*-regulatory elements in the upstream promoter region and regional epigenetic modifications, such as DNA methylation, histone modifications, and chromatin remodelling [35]. In addition to multiple promoters and promoter-like elements, it is now clear that bidirectionality is a common feature of promoters, with extensive analyses performed in yeast [97, 194] and human [97], with an estimated ~11 % of human genes expressed via bi-directional promoters. To date, the impact of this is not known, but it is suggested that bi-directional transcription has a role in maintaining an open chromatin structure at promoters, and may also provide a mechanism to spread the transcriptional regulatory signals locally in the genome or play a role in the coordinated expression of gene networks [194].

It is evident that eukaryotic promoters have evolved from the relatively simple “switches” found in bacteria, to the complex multi-factor regulatory regions found in mammals today. Complex promoters induce a range of responses to varying environmental conditions and cellular signals, facilitating controlled expression of the required gene variant according to developmental stage and cell type. Control of this kind is the basic requirement for producing the complex expression patterns necessary for cellular differentiation, and thus for the development of complex organisms.

## 5′ untranslated region

The 5′ untranslated region (UTR) is a regulatory region of DNA situated at the 5′ end of all protein-coding genes that is transcribed into mRNA but not translated into protein. 5′UTRs contain various regulatory elements (Fig. 1b) and play a major role in the control of translation initiation. Here, we discuss the regulatory roles of the 5′UTR, highlighting how the number and nature of regulatory elements present as well as the secondary structure of the mRNA and factor accessibility have major impacts on the expression of the downstream open reading frame [16].

### Structure

#### 5′cap structure

The 5′ cap is a modification added to the 5′ end of precursor mRNA that consists of 7-methylguanosine attached through a 5′-5′-triphosphate linkage (reviewed in [8]. This structure is essential for efficient translation of the mRNA, serving as a binding site for various eukaryotic initiation factors (eIFs) and promoting binding of 40S ribosomal subunits and other proteins that together make up the 43S pre-initiation complex (PIC) [74]. In addition to promoting translation, a recent study showed that the triphosphate linkage of the 5′ cap inhibits mRNA recruitment to the PIC in the absence of the full set of eIF factors [125]. The authors suggest that this mechanism allows inhibition of non-productive recruitment pathways, preventing the assembly of aberrant PICs that lack the factors required for efficient scanning and translation initiation [125]. The 5′ cap structure also functions in stabilisation of the mRNA, with various decapping enzymes acting to initiate decay from the 5′ end [123]. Although the major role of the 5′ cap seems to be the facilitation of mRNA translation, recent investigations of non-coding RNAs revealed that some types of non-coding RNAs, such as promoter-associated-RNAs (PASRs), are also capped [55]. The role of the cap in the regulation of these transcripts is currently unknown, and further studies are likely to reveal additional regulatory roles for this structure.

#### Secondary structure

The structure and nucleotide content of the 5′UTR appears to play an important role in regulating gene expression, with genome-wide studies revealing marked differences in structure and nucleotide content between housekeeping and developmental genes [61]. In general, 5′UTRs that enable efficient translation are short, have a low GC content, are relatively unstructured, and do not contain upstream AUG codons (uAUGs), as revealed by in silico comparisons of genes with low and high levels of protein output [86]. In comparison, 5′UTRs of genes with low protein output are, on average, longer, more GC rich, and possess a higher degree of predicted secondary structure [141]. These highly structured 5′UTRs are often associated with genes involved in developmental processes and the corresponding mRNAs are usually expressed in a developmental or tissue-specific manner. This variation in expression is likely to be mediated by interactions with different RNA binding proteins and structural motifs within the 5′UTR region. For example, the peroxisome proliferator-activated receptor γ (*PPAR*-γ) gene expresses a number of splice variants that differ in the 5′UTR rather than the protein-coding domain. Analysis of the translational activity of the various 5′UTRs found three that enhanced translation and two that had a repressive effect [115]. MFOLD modelling of mRNA folding in the 5′UTR revealed the presence of compact structures around the start codon in the repressive 5′UTRs. Although the exact mechanism of repression is unknown, it is likely that the differences in the structure and nucleotide content of the 5′UTRs facilitate binding of different proteins that act to either enhance or repress translation.

A well-characterised secondary structure that has a major impact on translation is the G-quadruplex structure (G4). These structures are guanine-rich nucleic acid sequences that can fold into a non-canonical tetrahelical structure that is very stable and has the ability to strongly repress translation [11]. Bioinformatic studies have shown that these structures are often highly conserved, can be found in regulatory elements other than the 5′UTR such as promoters, telomeres and 3′UTRs, and are enriched in mRNAs encoding proteins involved in translational regulation and developmental processes, indicating that they are an integral part of various important biological processes [11]. Many G4 structures have also been found in oncogenes. The *TRF2* gene, which is involved in control of telomere function, has a G-rich sequence within its 5′UTR that can fold into a G4 structure and repress translation of a reporter gene by 2.8-fold [65]. This gene is overexpressed in a number of cancers, indicating that the G4 is in place to tightly regulate the expression of this gene. Gomez and colleagues also demonstrated that a number of ligands that bind to G4 structures were able to modulate the translation efficiency of *TRF2* in vitro [65]. In conclusion, G4s appear to have a major impact on the translational regulation of the genes in which they reside [11] and may repress translation by secondary structure alone or by modulating interactions with proteins and other factors.

The scanning model of translation initiation proposes that upon binding to the 5′ cap the 43S ribosome complex scans the 5′UTR until it locates the optimal AUG codon and initiates translation [88]. This model led to an assumption that all mRNAs with highly structured 5′UTRs have low translation rates due to inability of the ribosome to scan through tight secondary structures such as stem-loops. However, some recent studies have shown that this is not the case. Firstly, a report [42] highlighted the limitations of the previously preferred analysis method used by many groups, the rabbit reticulocyte lysate (RRL) system [139]. In a comparison of methods for studying translation, they found the RRL system possessed a number of flaws, the most important of which was that capping did not seem to significantly affect translation when using this cell-free system. As it is well established that the 5′ cap is essential for efficient translation, and that the effect of the 5′ cap is much more pronounced for some mRNAs compared to others, the RRL system seems not to reflect in vivo conditions [160]. In addition, correlating evidence from experiments using a different cell-free system (wheat germ S30 system) and cultured cells demonstrated that capping increased the translational efficiency for most RNAs by several orders of magnitude [42]. Importantly, using these two systems, Dmitriev found that there was no dramatic difference in the translational efficiency between a number of short unstructured and longer highly structured 5′UTRs that they examined in their study. These data indicate that the natural stem-loop structures in these 5′UTRs do not seem to inhibit initiation. Despite this, large-scale in silico studies have shown there is a significant correlation between 5′UTR folding free energy and protein abundance [153]. This does not mean that the structure itself is the inhibitory factor, although it does suggest that 5′UTR secondary structure is involved in post-transcriptional regulation. It has been emphasised that interactions with RNA-binding proteins prior to scanning and initiation are likely to affect the mechanism of searching for the initiator codon [42]. For example, the eIF4F complex assembles on the 5′ cap prior to translation and unwinds secondary structures in the 5′UTR in order to promote loading of the 43S ribosomal complex onto the mRNA [81]. This correlates with the results obtained by Dmitriev and also helps explain why direct inhibition via secondary structures is observed in the RRL system, as this system has a highly reduced content of mRNA-binding proteins [172]. The human L1 bicistronic mRNA contains a 900-nt-long 5′UTR with high GC content (~60 %) and two short upstream open reading frames (uORFs). Predicted folding reveals a number of potential stem-loop structures; however, the L1 mRNA is still translated very efficiently via cap-dependent initiation [43]. The above examples provide strong evidence that the unwinding of stem-loops occurs sequentially and indicate that the current practice of using in silico predictions of folding energies of 5′UTRs to forecast translatability is likely to result in incorrect assumptions.

#### Alternative 5′UTRs

In addition to those UTRs generated via the use of alternative promoters, alternative 5′UTRs may be produced by alternative splicing or through variation of the transcription start site from a single promoter [163]. Diversity within the 5′UTR of a gene enables variation in expression, depending upon the nature of the regulatory elements contained within each alternative 5′UTR. Slight changes in the arrangement of translational control elements between isoforms can lead to major changes in the regulatory effects on translation [151]. A large-scale analysis of the mammalian transcriptome indicates that expression of alternative 5′UTRs is a widespread phenomenon, with most genes having the potential for differential expression [73]. Genes that are known to consistently express multiple 5′UTRs are typically involved in functional activities such as transcription and other signalling pathways [151]. The oestrogen receptor β gene (*ERβ*) plays an important role in oestrogen function and the expression of its multiple isoforms is frequently mis-regulated in cancers. Smith and colleagues have recently identified three alternative 5′UTRs (termed UTR a, c and E1) that contribute to the expression of the different isoforms [164, 165]. They found that UTRs a and c inhibited translation, with UTRa having a very potent inhibitory effect, while E1 had a less pronounced, but still inhibitory, effect, despite being only 90 nt long and having low predicted secondary structure. The expression of alternative 5′UTRs represents an evolutionary gain of transcriptional and translational control pathways, allowing tissue-specific expression patterns and expanding the repertoire of expression from a single gene locus.

### Regulatory motifs

The lack of correlation between the rate of translation and the length or structure of the 5′UTR in both capped and uncapped mRNAs, as well as the ability of certain genes to be expressed under conditions of stress indicates that there must be other elements within eukaryotic mRNAs that contribute to translation initiation and control of gene expression via the 5′UTR.

#### IRES and cap-independent translation initiation

Internal ribosome entry sites [14] are mRNA regulatory motifs that facilitate a cap-independent mechanism of translation initiation, in which the ribosome binds to an internal site close to the translation initiation site [118]. IRES allow recruitment of ribosomes to capped or uncapped mRNAs under conditions when cap-dependent translation is inhibited by stress, cell-cycle stage or apoptosis, ensuring the continued expression of essential proteins required for cell function. A number of IRES-containing genes such as *c*-*Myc*, *Apaf*-*1* and *Bcl*-*2* are required at low levels during normal cellular growth, but are induced via the IRES pathway under conditions of stress [87]. It is thought the IRES pathway may also contribute to maintaining the low expression levels required under normal cellular conditions by sequestering ribosomes and reducing their binding at the main translation initiation site. The mechanism of internal initiation is still poorly understood, although it is clear that efficiency of IRES is heavily reliant upon *trans*-acting protein factors, allowing cell-specific IRES-mediated translation of mRNAs [141].

Structures in the 5′UTR have been shown to influence IRES activity, which may occur via interactions with various trans-acting factors, or by direct interactions with ribosomes. An example of genes in which IRES activity is regulated by *trans*-acting factors is the Myc family of proto-oncogenes that are involved in cell proliferation. Recruitment of ribosomes to the IRES is dependent upon at least four proteins that bind and alter the conformation of the mRNAs to allow interaction with the 40S subunit [33]. Another example is the Hepatitis C virus (*HCV*), containing a highly structured IRES that initiates cap-independent translation via two major structural domains, consisting of conserved stem-loop structures that interact with the 40S ribosomal subunit to form a complex and recruit eIF3 [100]. The structures of eukaryotic IRES are very diverse and no universally conserved sequences or structural motifs have yet been identified. For some genes, specific and stable RNA structures are required for efficient IRES activity, while in other genes, stable structure is inhibitory to IRES-mediated translation [57]. It has been suggested that IRES are not rigid structures but can undergo transitions that substantially influence their activity [87]. IRES elements may also result in the production of different protein isoforms, thus expanding the repertoire of expression from a single gene [87].

The presence of IRES between different AUG and non-AUG initiation codons suggests a role for IRES in promoting translation initiation from weak alternative start codons [179]. IRES may also interact with uORFs, another class of regulatory elements discussed in the next section. Gilbert [64] discusses recent findings on IRES and draws attention to flaws in the methods for defining IRES (bicistronic test) that may result in false positive predictions [64]. Although IRES are an important mechanism for some genes, Gilbert suggests that it is wrong to assume the presence or activity of an IRES by prediction alone, emphasising the importance of experimental validation. IRES are a poorly understood but important regulatory mechanism, and further investigation will be needed to discern the mechanisms and context of initiation via IRES.

#### uORFs

Upstream open reading frames occur in 5′UTRs when there is an in-frame stop codon following an upstream AUG (uAUG) codon, prior to the main start codon (reviewed in [124, 126, 189]. uORFs are present in ~50 % of human 5′UTRs, and their presence correlates with reduced protein expression and with mutation studies indicating that, on average, uORFs reduce mRNA levels by 30 % and reduce protein expression by 30–80 % [17]. Ribosomes binding to an uAUG may translate an uORF, which can impact on downstream expression by altering the efficiency of translation or initiation at the main ORF. If efficient ribosome binding does not occur, the result will be a reduction of protein expression from the gene. Alternatively, synthesis may continue from the uORF and produce an extended protein that may be detrimental. Decreased translational efficiency is a well-characterised effect of uORFs within a 5′UTR [126], illustrated by the poly(A)polymerase-α (*PAPOLA*) gene that contains two highly conserved uORFs in the 5′UTR. Mutation of the 5′ proximal uAUG codon resulted in increased translation efficiency, indicating that the uORF has a significant inhibitory effect on the expression of this gene [149]. It is commonly thought that uORFs decrease translational efficiency by rendering the ribosome unable to reinitiate translation following termination from the uORF [118]. However, a recent study of over 500 uORF-containing gene loci found no significant correlation between the impact of the uORF on the expression of the downstream gene and the distance between the uORF and the coding sequence (CDS) [17]. The authors suggest that it is likely that, in genes containing a single uORF, CDS translation occurs from ribosomes that scan through the uORF, rather than via re-initiation. This is in contrast to the work of Kozak [88], and the general consensus on uORFs. To further complicate matters, experiments using cells depleted of Rent1, a factor involved in nonsense-mediated decay (NMD), revealed that, in the absence of NMD, transcripts containing uORFs were generally upregulated [120]. This implies that NMD also plays an important role in the regulation of these transcripts. The results from these studies indicate that the mechanism of uORF gene knockdown is more complex than the scanning model proposes, and that further experimental work will be required to elucidate this mechanism.

AUG codon recognition is influenced by a number of factors, including proximity of the AUG to the 5′ cap, the flanking sequence and secondary structure [90]. uORFs appear to exist as regulatory elements that act to control the translation of the downstream ORF. Protein kinase C (*PKC*) represents a family of serine/threonine kinases that play a major role in the regulation of cell growth and differentiation [150]. The novel PKCη isoform has a specific tissue distribution and is primarily expressed in cells undergoing high turnover, such as epithelial cells. Recent studies found that this isoform has a special role in the response to stress and its expression has been found to correlate with drug resistance in various cancer types [156]. The 5′UTR of human *PKC*η is long (659nt), GC rich, and contains two small conserved uORFs [150]. Mutations introduced into each of the uORFs resulted in modest increases in expression (1.5- and 2.2-fold increases) and a double mutation resulted in a 3-fold increase in gene expression from the main AUG. This mechanism of translational repression is likely to be in place to control the expression of *PKC*η under normal cellular conditions [150]. Under conditions of stress, the two uORFs also play a role in expression as they facilitate leaky scanning to enhance the translation of the main ORF. Varying levels of ribosome binding and translation of each of the uORFs may also contribute to cell-specific “tweaking” of gene expression.

Despite the majority of uORFs having a negative impact on gene expression, there are some cases in which the presence of a uORF actually enhances translation. Bicistronic *vpu*-*env* mRNAs are involved in HIV-1 virus expression, and they contain a conserved minimal uORF [90]. This uORF is only 5 nt upstream of the *vpu* AUG and is immediately followed by a termination codon that overlaps the main AUG. Krummheuer and colleagues showed that this uORF has a significant positive impact on the translation of *Env* while not interfering with translation of *Vpu* [90]. Mutants in which the distance between the uORF and the main AUG was increased by five codons indicated that the uORF is not involved in the initiation of *Vpu*, and the authors suggested that the minimal uORF may act as a site for ribosome pausing, allowing it to interact with an RNA structure that supports a ribosome shunt, a process during which the ribosome physically bypasses part of the 5′UTR to reach the initiation codon.

The role of uORFs as regulatory elements acting on the process of ribosome binding and translation is well studied, but the function or fate of the encoded peptides is often unknown, perhaps due to the difficulty in analysing the expression levels and localisation of the peptides. Evidence that peptides translated from uORFs are present in cells was first shown by Oyama and colleagues, who identified 54 proteins of <100 amino acids expressed in human chronic myelogenous leukemic cells that were all mapped back to uORFs [135]. Although proteins were identified, thousands of uORFs did not seem to produce a detectable protein product in these cells, which indicates that either (1) proteins derived from uORFs may be selectively proteolysed in the cells, (2) some of the uORFs are expressed but not in this cell type, or (3) many do not produce proteins. Despite this, it is clear that some uORFs do produce peptides that are retained in the cell and thus are likely to be functional, although to date there are no comprehensive studies on the function of proteins translated from an uORF.

The past decade has revealed that regulation via uORFs is a complex process that acts to tightly regulate the expression of the genes they control. A good example of complex control of gene expression via uORFs was outlined recently [171]. RNase H1 is present in the nuclei and mitochondria of mammalian cells and is differentially expressed among cell types. Two different in-frame AUGs control the expression of these isoforms and an uORF is also present in the 5′UTR of this gene. Experimentation revealed that translation of the mitochondrial RNAse H1 is initiated at the first AUG, which is restricted by an uORF, resulting in the mitochondrial isoform being about 10 % of the abundant of the nuclear form [171]. Translation of the nuclear isoform proceeds from the second AUG and is unaffected by the presence of the uORF, as the ribosome either efficiently reinitiates or skips both the first AUG and the uORF. This regulation allows control of RNase H1 expression in mitochondria, where its excess or absence can lead to cell death, without affecting the normal expression levels of the nuclear isoform. Suzuki and colleagues also found that altering the context of the AUG altered transcript accumulation, meaning there must be other factors involved. This example illustrates the combinatorial use of multiple uORFs and other factors to produce a highly specific system of translational regulation. In addition, alternative promoters or splicing, as well as the finding that out-of-frame and sub-optimal initiation codons can, in certain contexts be available to ribosomes, and are all factors that can affect uORF expression, further increasing the diversity of regulation and translation emerging from these regions [136].

Mutations involving uORFs are likely to be detrimental, as they can disrupt the control of gene expression, resulting in aberrant gene expression levels that may subsequently lead to disease [26]. Mutations disrupting the uORF in the 5′UTR of the gene encoding the human hairless homolog (*HR*) and resulting in increased translation of the gene, have been associated with Marie Unna hereditary hypotrichosis, an autosomal dominant form of genetic hair loss [188]. Mutations that create novel uORFs may also have a detrimental effect by interfering with normal expression. It has been speculated that a mutation in a tumour suppressor gene may result in decreased production of protective proteins and contribute to the onset of cancer [189]. These examples illustrate the importance of uORFs in the control of specific gene expression and in maintaining homeostasis, and variability within uORFs is thought to contribute to individual phenotype and disease susceptibility [189].

### Conclusions

Disease-causing mutations situated within 5′UTRs confirm the importance of motifs in gene expression and regulation. The ferritin 5′UTR contains a stem-loop structure called an iron response element, and mutations in this region have been associated with hereditary hyperferritinemia cataract syndrome. It is likely that mutations within the stem-loop alter the structure, resulting in abnormal processing of iron and manifestation of disease [26]. Regulation mediated by 5′UTRs involves the combinatorial effects of a multitude of factors and relies heavily on the secondary structure and accessibility of protein binding sites. In addition to the regulatory elements outlined above, it is likely that future investigation will reveal novel factors that interact with the 5′UTR, prior to translation, and influence gene expression.

## Intronic regions

Introns are regions of DNA that are transcribed into pre-messenger RNA but are removed during splicing to generate a mature mRNA. Spliceosomal introns are present in all studied eukaryotic organisms. The exact origin of introns is debated, but it is widely accepted that introns evolved soon after the divergence of prokaryotic and eukaryotic organisms and that the current intron content of any particular genome is the result of both intron loss and gain over time (for thoughts and reviews on the topic of intron evolution, see [103, 154]. Regardless of when and how introns arose, it is clear that the appearance of introns was an important catalyst for evolution, facilitating rapid evolution at the protein level through increased rates of meiotic crossing over within coding regions, as well as rapid evolution of regulatory elements due to relaxed sequence constraints within non-coding introns [54]. Introns would also have allowed evolution of RNA regulatory pathways without interfering with protein expression, an important distinction that was only made possible by the separation of transcription and translation [103].

### Organisation and length

Intron organisation, position and length may influence the ability of the intron to affect gene expression. Intron content varies between different species and some eukaryotic lineages maintain numerous large introns while others seem to have undergone intron loss throughout evolution [154]. The average human gene contains 5–6 introns with an average length of 2,100 nt [54], although extremes at either end of the spectrum exist. In humans and other animals, intron length is, in general, inversely correlated with transcript levels. A cross-species comparison between yeast, *Arabidopsis* and mouse found that genes involved in stress-response, cell proliferation, differentiation or development generally showed significantly lower intron densities than genes with other functions [75]. Genes in these categories require rapid regulation in response to changing conditions, suggesting that introns may be detrimental to this process. Organisms with short generation times were also found to have a significantly lower genome-wide intron density. Through comparison between the three model organisms, Jeffares and colleagues observed that mouse genes seem to be comparatively less optimised for rapid regulation (i.e. they have higher intron densities), which is logical as mammals are less exposed to rapid environmental changes than plants and microorganisms [75].

Introns of very different lengths are often found within a gene, although to date, there are no data indicating a global trend concerning length and position except for the first intron. A large-scale comparison of intron lengths relative to their position in the gene found that the first intron of the CDS tends to be ~40 % longer than later introns [16]. Significantly longer first introns were found in species from diverse phylogenetic groups (including vertebrates, insects, plants and fungi), suggesting that this increased length is a common feature of genes in all eukaryotic species. This study also revealed that the first intron was longer again in genes that did not contain an intron within the 5′UTR. In addition to the length of the first intron, a large-scale bioinformatic study that examined 18,217 human ref-sequence genes found these introns, particularly in the first 100 bp, to be enriched for G-rich regions that have the potential to form G4s [48]. G4 structures have significant negative effects on translation when located within the 5′UTR of a gene. G-rich elements in the first intron may provide structural targets for regulatory proteins and have an effect on transcription or RNA processing. The position of the first intron relative to the promoter and translation start site means it is a region in which regulatory elements are likely to evolve, as elements within this region are more likely to have a significant effect on promoter activity than elements situated further downstream. In addition, evolution of regulatory elements can occur without disrupting the coding sequence. It is thus likely that the increased relative length of the first intron in many genes is the result of the evolution of regulatory elements (including G4s) within this region.

### Introns in the UTRs

A genome-wide functional analysis of the 5′UTRs of human genes found that approximately 35 % of human genes contain introns in the 5′UTR [21]. 5′UTR introns were found to differ from introns within coding regions with respect to nucleotide composition, length and density, with 5′UTR introns found to be on average twice as long as those in coding regions and generally lower in density. Interestingly, the results from this comprehensive study indicated that the most highly expressed genes tended to have short rather than long 5′UTR introns or lacked them entirely [21]. Genes with regulatory roles were also enriched for 5′UTR introns, providing further evidence that the presence of at least one intron within the 5′UTR enhances gene expression either by enhancing transcription or stabilising the mature mRNAs. An intron in the 5′UTR may enhance gene expression through the presence of transcriptional regulatory elements, or through structural modulation and splicing. For example, expression of the ubiquitin C (*UbC*) gene is dependent on the presence of an intron in the 5′UTR. Deletion analyses showed that promoter activity is significantly reduced when the intron is removed, and electrophorectic mobility shift and supershift assays demonstrated that both Sp1 and Sp3 transcription factors bind this region at multiple sites [13]. These experiments indicate that elements within the intron play a major role in the transcriptional regulation of this gene.

In contrast to 5′UTRs, 3′UTRs were found to have relatively few introns (5 %) [21]. A study looking at rare cases of intron acquisition in retroposed mammalian genes found that the presence of an intron in the 3′UTR of these genes resulted in down-regulation of gene expression by nonsense-mediated decay [52]. This negative effect on expression offers an explanation for the low prevalence of 3′UTR introns. In addition, an in silico study analysing the effect of retained 3′UTR introns upon miRNA target sites indicated that some transcripts only contain miRNA binding sites if the intron in the 3′UTR is retained [174]. This suggests that variations in intronic splicing in the 3′UTR could result in isoform-specific regulation via miRNAs that may be utilised in a tissue-specific manner.

### Intron function

Introns could have deleterious effects on gene expression, such as a delay in mature transcript production due to splicing or increased pre-mRNA length, and the energy required to produce a transcript containing introns is also substantially higher. However, the high prevalence of introns in eukaryotic genomes indicates that the benefit must outweigh the potential negative effects. Introns function in a number of different ways and are

- sources of non-coding RNA;
- carriers of transcriptional regulatory elements;
- contributors to alternative splicing;
- enhancers of meiotic crossing over within coding sequences and thus drivers of evolution;
- signals for mRNA export from the nucleus and nonsense-mediated decay [53].

The effect of introns on genome evolution has already been discussed, but introns also have an important role in the regulation of gene expression, as demonstrated by experiments in which introns are removed or in which introns were inserted into transgenes, resulting in enhanced expression (for an example, see [25]. Indeed, many genes with an intact promoter are essentially not expressed at all in the absence of an intron, demonstrating the relative importance of the intronic and promoter regions in some genes [155]. Introns can enhance gene expression through the presence of transcriptional enhancers or alternative promoters, or by a less well-understood mechanism termed intron-mediated enhancement that arises from introns and increases the processivity of the transcription machinery at the elongation stage. By this mechanism, introns ensure efficient completion of transcription of the gene and could also reduce transcription from sequences that are not genuine promoters [155]. As well as containing regulatory elements, introns are characterised by a significantly lower nucleosome density in comparison to exons [130], and different histone modifications define exons, alternatively spliced exons, and introns [37].

### Regulatory elements

#### Enhancers

Enhancers are segments of DNA that enhance transcription of genes by interactions with *trans*-acting factors. Enhancers generally interact in a specific manner with the corresponding promoter through chromatin looping of the intervening DNA, to associate enhancer-bound transcription factors with the promoter [131], and recent data have indicated that enhancers may also affect downstream processes, such as decompaction of the chromatin fibre and the release of RNAPII [133]. Although these elements interact specifically with the promoter, enhancers are variable, and upstream, downstream and distal elements have been identified that can activate transcription, independent of their location or orientation with respect to the promoter [133]. Enhancers are now recognised as the main regulatory elements involved in transcription and many enhancer elements are critical in defining the expression patterns of genes. An enhancer element situated within an AT-rich regulatory region in the first intron of *Imp2* is critical for the expression of this gene. This enhancer serves as a binding site for HMGA2 that acts to recruit and stabilise a complex of transcription factors, resulting in *Imp2* transcription [32]. Mutations that disrupt enhancer activity may also have a profound effect on the expression of the downstream gene. Enhancer activity in the *OCA2* gene is strongly associated with variation in human eye colour [45]. SNPs disrupting a conserved enhancer that binds helicase-like transcription factor (HLTF) upstream of this gene reduce the expression and result in blue eye colour, with a frequency of 78 % [168]. This emphasises the importance of many enhancers in regulating gene expression and provides evidence that variations within enhancers are likely to contribute to individual phenotype and disease susceptibility.

Recent studies using genome-wide tools have indicated that many enhancers are associated with specific histone modifications, that allow them to be recognised and utilised in a specific manner [133]. Promoters can generally be influenced by distinct enhancer elements under varying conditions [102], while binding of factors that do not associate strongly with the promoter may “switch off” the enhancer as required. An enhancer region that is critical for specific gene expression during development is the human-accelerated conserved non-coding sequence 1 (*HACNS1*). This element is the most rapidly evolving human non-coding element identified to date and experiments using a transgenic mouse model showed that this element drove strong and specific reporter gene expression in the anterior limb bud, pharyngeal arches, and developing ear and eye, indicating that *HACNS1* acts as a robust enhancer during development [146]. In contrast, the chimpanzee orthologue failed to drive reproducible reporter gene expression in a similar manner, suggesting that this region is vital for development of human-specific digit and limb patterning that distinguishes humans from other primates, specifically bipedialism and dexterity of the human hand.

The complexity arising from enhancers is increased by the fact that often multiple enhancers and other elements interact and have a combinatorial effect on gene expression. The cystic fibrosis transmembrane conductance regulator (*CFTR*) gene is activated by coordinated regulation from several intronic enhancers that bind both tissue-specific and general transcription factors [134]. Differential interactions between the various enhancers and the promoter were found to result in variable expression levels in epithelial cells of intestinal lineage (high expression) and of the respiratory system (lower expression) and chromatin conformation capture was used to identify distal regulatory sites that also contributed to gene expression. This example shows how complex interactions between enhancers and distal elements can contribute to the tissue-specific expression of a gene. In addition to controlling the differential expression of a single gene, conserved enhancers have been found that contribute to the regulation of whole gene pathways. Transcription factor Ronin and the transcriptional coregulator Hcf-1 are essential factors involved in the self-renewal of embryonic stem (ES) cells. They bind to a highly conserved enhancer element in a subset of genes that function in transcription initiation, mRNA splicing and cell metabolism [36]. The enhancers that bind Ronin/Hcf-1 are thus key elements required for ES cell pluripotency.

In vivo analyses of evolutionarily conserved non-coding sequences revealed an enrichment of developmentally specific *cis*-regulatory transcriptional enhancers [146]. Indeed, the high proportion of non-coding to coding regions in the human genome compared to other species provides strong evidence that the complexity of humans arises from evolution of these non-coding regions, with enhancers likely playing a major role in this process.

## 3′ untranslated region

The 3′ untranslated region (3′UTR), situated downstream of the protein coding sequence, has been found to be involved in numerous regulatory processes including transcript cleavage, stability and polyadenylation, translation and mRNA localisation. They are thus critical in determining the fate of an mRNA. In comparison to the 5′UTR, which contains sequences responsible for translation initiation, sequence constraints within the 3′UTR are more relaxed resulting in a greater potential for evolution of regulatory elements. Despite this, regions of high conservation are also prevalent, with 3′UTRs containing some of the most conserved elements within the mammalian genome [161]. A genome-wide in silico analysis revealed that contrary to the promoter region, motifs in the 3′UTR are primarily conserved on one strand, which is consistent with the 3′UTR acting to regulate gene expression at the post-transcriptional level [193]. The 3′UTR serves as a binding site for numerous regulatory proteins as well as microRNAs (Fig. 1c), and in order to understand the properties of this region, it is necessary to first discuss the research history of these interactions.

### MicroRNAs and the 3′UTR

MicroRNAs (miRNAs) are endogenous, single-stranded non-coding RNA molecules of ~22 nt in length that interact with mRNA targets post-transcriptionally to regulate expression. In animals, miRNAs generally exert an effect by partial base pairing to a miRNA response element (MRE) on a target mRNA via a ‘seed sequence’ at the 5′ end of the miRNA, which then recruits Argonaut and inhibits translation of the mRNA (see [62, 137, 166]. Another mechanism by which miRNAs can down-regulate genes is through perfect base pairing with a target sequence, promoting RNA cleavage, although only a few examples of this have been described [195]. In addition to down-regulating gene expression, some miRNAs, such as the tumour necrosis factor-alpha and the cytoplasmic beta-actin gene, have been found to induce translational up-regulation [63, 182]. Data indicate that miRNA repression occurs in proliferating cells, while activation is mediated by some miRNAs during cell cycle arrest [128, 182]. miRNAs are the most extensively studied group of non-coding RNAs and interested readers are referred to current reviews on miRNA functions and mechanisms [51, 72, 76], miRNA response element prediction [157], miRNA-mediated regulation of developmental processes [190, 198], regulation of miRNA expression [89] and the impact of miRNAs on evolution of 3′UTRs [197].

A wealth of information regarding miRNA expression and function is now available, and it is evident that miRNAs are a vital component of gene control. miRNAs have been found to be involved in most important biological events including cell proliferation and differentiation, development, nervous system regulation and tumourigenesis (reviewed in [72], and common miRNA targets include transcription factors and signalling proteins [197]. An individual miRNA has the ability to regulate a large number of target genes because complementarity is only required in the seed region, and miRNAs may be involved in the regulation of a process or system. In addition, an mRNA may be regulated by multiple different miRNAs, expanding the repertoire of expression of an mRNA at a given time, in a particular cell type. Studies on MRE prediction and validation have shown that the presence of multiple seed sequences within an mRNA is common (~50 % of targets) and targets are frequently expressed in a mutually exclusive manner to the miRNA, further indicating a role for miRNAs in fine-tuning of gene expression and developmental processes [167]. miRNAs may also interact with various RNA binding proteins to mediate efficient and precise cellular responses to various signals and changing conditions. Trisomy 21, the cause of Down syndrome, has a severe and complex phenotype. In silico analysis has shown that five miRNA genes are duplicated in this event, and overexpression of these genes has been proposed to reduce the expression of target genes, contributing to the severe phenotype of this syndrome [50].

Many miRNAs are evolutionarily conserved [10, 198], and the lack of requirement for long regions of complementarity means that novel miRNAs and MREs can easily arise, implicating them as powerful tools for evolution [167]. miRNAs bind preferentially in the 3′UTRs of protein-coding genes, although some target sites have been identified in the 5′UTR and intronic gene regions. An inter-species genome-wide comparison found that motifs in the 3′UTR are an average of 8 bp in length and that around half of all the motifs identified are likely to be related to miRNAs [193]. miRNAs are often expressed in a tissue-specific or developmental stage-specific manner, and genes involved in processes common to all cells have evolved to selectively avoid sequences complementary to miRNA seed regions [167]. This mechanism of selective avoidance has a significant impact on the evolution of the 3′UTR. A recent study found that modification of the stop codon to extend the coding region of a transgene reporter changed the mechanism from miRNA-induced translational repression to RISC-mediated degradation by small interfering RNAs [69]. These results indicate that active translation impedes miRNA-RISC interaction with target mRNAs and provides an explanation as to why MREs are contained in the non-coding regions. Data obtained in vitro and in vivo supported the conclusion that, while siRNA can work efficiently in non-coding and coding regions, miRNA activity is significantly inhibited when targeting the coding region, indicating that miRNA-programmed RISC is required to remain attached to the target mRNA to effectively silence translation in *cis* [69]. Data also provided a possible explanation for the low prevalence of MREs situated in the 5′UTR, as scanning of the 5′UTR by the translation initiation complex may impair formation of miRNA-RISC complexes.

### Stabilisation and AU-rich elements

Modification of transcript stability allows expression to be rapidly controlled without altering translation rates. This mechanism has been found to be critically involved in vital processes such as cell growth and differentiation, as well as adaptation to external stimuli [46, 49]. The most well-characterised stabilisation elements are the AU-rich elements [75] that are situated in the 3′UTR of some genes. These elements range in size from 50 to 150 bp and generally contain multiple copies of the pentanucleotide AUUUA [27]. AREs play a critical role in the stability of particular genes. Early studies indicated that AREs are variable in sequence and three main classes have been defined that differ in the number and arrangement of motifs, where class III contains no AUUUA motifs (reviewed in [124]. AREs bind proteins (ARE-BPs) that generally promote the decay of the mRNA in response to a variety of intra- and extra-cellular signals (for some recent examples, see [23, 85, 92], although binding proteins that act to regulate translation have also been described [98]. Genes regulated by AREs include cytokines, growth factors, tumour suppressors and proto-oncogenes, as well as genes involved in the regulation of the cell cycle, such as cyclins, enzymes, transcription factors, receptors and membrane proteins [46]. This plethora of vital gene families affirms the significance of transcript stability in the process of gene regulation.

Many ARE-BPs are expressed in a tissue- or cell-type-specific manner [152], with ARE secondary structure being an important factor in ARE-BP activity [119]. Different ARE-BPs can compete for the same binding site and, depending on the cellular localisation, environment and timing, regulation from an ARE can result in different outcomes for a transcript. A class III ARE in the *c*-*jun* 3′UTR has been shown to decrease steady-state mRNA levels and also be involved in increasing protein production [9]. This seems counterintuitive, but it is likely that each mechanism is used at different times for different needs, such as in developmentally or tissue-specific circumstances. Environmental factors can also impact ARE protein binding, with stability playing a major role in response to stresses such as heat shock and nutrient deprivation. These stimuli trigger a signalling cascade that alters the abundance of various ARE binding proteins, while simultaneously manipulating RNA binding properties (reviewed in [46]. Expression of the anti-apoptotic protein Bcl-X_L_ is increased by stabilisation following UVA irradiation, a process implicated in skin and other cancers. Examination of the ARE-BPs associated with an ARE in the *Bcl*-*X*_*L*_ 3′UTR identified nucleolin as a key stabilising protein, and the authors suggest that UVA irradiation increases the binding capacity of nucleolin to the ARE and facilitates protection of the *Bcl*-*X*_*L*_ mRNA from degradation [196].

In addition to affecting stability, AREs have also been found to activate translation, although this pathway is less common and is poorly understood. For example, the 3′UTR of cytokine tumour necrosis factor α (TNFα) mRNA contains a highly conserved 34nt ARE [181]. This gene is expressed in stimulated lymphocytes and is critical for inflammatory response so must be rapidly regulated when required. During inflammation, cell growth is arrested and up-regulation of TNFα occurs at the protein level. Studies found that Argonaut 2 (AGO2) and fragile-X mental retardation syndrome-related protein 1 (FXR1) associate with the ARE of *TNFα* and function to activate translation in response to serum starvation [181]. It was also found that human miR369-3 binds through the seed sequence to the ARE and directs association of these factors with the ARE to activate translation, providing evidence for a secondary role of miRNAs in translation, alongside their well-studied destabilising roles [182]. An earlier study examining the structure of the *TNFα* ARE showed that hairpin folding modulates binding of proteins to that motif and mediates different outcomes for the mRNA [56]. These experiments demonstrate the versatility of AREs, RNA-binding proteins and miRNAs in modulating gene expression in a positive or negative manner, as required. The ability of AREs to influence both mRNA stability and translation is likely to result from different signals received. The GU-rich element (GRE) is another recently discovered stability element that interacts with CUGBP1, an RNA binding protein that promotes the decay of the associated mRNA [94, 184]. Alongside microRNAs, AREs and GREs have impacted upon the evolution of the 3′UTR, and thus shaped the regulation of gene expression from this region.

### Structure

#### Poly(A) tail

The poly(A) tail results from the addition of a series of adenosine bases to the 3′ end of an RNA molecule. This provides the mRNA with a binding site for a class of regulatory factors called the poly(A) binding proteins (PABP) that have roles in the regulation of gene expression, including mRNA export, stability and decay and translation (reviewed in [67, 101], playing vital roles during vertebrate development [68]. Five different PABPs have been identified in humans (one nuclear and four cytoplasmic), all of which have distinct functional roles [68]. PABPs seem to function as scaffolds for the binding of numerous other factors, thus they indirectly regulate gene expression. Aside from their global effects on translation, PABPs can also regulate the translation of individual mRNAs, although this is less well documented (e.g. Cyclin B [18]). PABP mRNAs can also bind poly(A) tracts in their own 5′UTRs, repressing their own translation and maintaining balance and controlled regulation. The poly(A) tail is synthesised at a defined length (~250 bp in mammalian cells), which may then be shortened in the cytoplasm to promote translational repression as required [91].

#### 5′–3′ interactions

Early experiments investigating the roles of the 5′cap structure and the poly-A tail found that they function synergistically to control mRNA translation [60]. The addition of a poly(A) tail to a luciferase reporter gene increased protein expression 97-fold when the length of the 3′UTR was 19 bases [175], demonstrating the essential role of the poly(A) tail in efficient translation. The association of PABPs with the poly(A) tail facilitates an interaction with eIF4F bound to the 5′cap structure, resulting in circularisation of the mRNA that promotes translation initiation and ensures ribosome recycling and efficient translation (for reviews on translation initiation and the 5′–3′ interaction pathway, see [28, 74, 114]. This interaction also allows inhibition of translation by inhibitor proteins bound to the 3′UTR, which is important because the relative lack of constraint in RNA secondary structure in the 3′UTR compared to the 5′UTR indicates that response to changing conditions can occur with less consequences while feeding back information to the initiation site [114]. In addition to binding through protein interactions at the 5′cap structure, sequence specific interactions between the 5′ and 3′ ends of an mRNA have also been observed. The human p53 gene contains a region of complementarity between the 5′ and 3′UTRs that have been shown to interact and bind translation factor RPL26 that mediates translational up-regulation as a response to DNA damage [28]. Mutations affecting the termination codon, poly-adenylation signal and secondary structure of a 3′UTR can cause translation de-regulation and disease [26].

A genome-wide analysis of UTRs identified numerous motifs within human 5′UTRs that were specific to the 3′ ends of miRNAs, with many of these found to simultaneously contain 5′ end interaction sites in the 3′UTRs [93]. Further investigation demonstrated interactions between the 5′ and 3′ ends of many genes are facilitated by an interaction with a single miRNA, and that genes highly influenced by miRNA overexpression or deletion contained predicted binding sites in both UTRs. The authors termed this class of miRNA targets miBridge, and reporter gene assays revealed that deletion of either binding site reduced repression from the miRNAs, indicating that the interaction is essential for potent down-regulation of the transcript [93]. It is clear that interactions between the 5′ and 3′UTR contribute to the precise control of expression pathways and responses, and mRNA circularisation provides an explanation as to how translation can be so efficiently repressed via protein or miRNA binding in the 3′UTR.

#### Length

The requirement of 5′–3′ interactions for efficient translation has implications for both the length and secondary structure of the 3′UTR, with studies demonstrating the significant impact of some longer 3′UTRs on expression. Using a luciferase reporter gene, Tanguay and Gallie [175] observed that increasing the length of the 3′UTR from 19 to 156 nt decreased expression ~45-fold, independently of the orientation, gene or sequence [175]. This early example indicates 3′UTR length is a major determinant in mRNA expression. Aside from the importance of interaction with the 5′UTR, the prevalence of miRNA binding sites also has an impact on the length, as longer 3′UTRs are more likely to possess miRNA binding sites that have the potential to inhibit translation. A study comparing the length and miRNA-binding site content of ribosomal and neurogenesis genes found that ribosomal genes had shorter 3′UTRs and specifically avoided miRNA-binding sites, when compared to random controls [167]. In contrast, 3′UTRs of genes involved in neurogenesis were longer and specifically enriched for potential binding sites. The *Hip2* gene uses alternative 3′UTRs to control expression as required. The longer 3′UTR of this gene contains conserved seed matches to two miRNAs that are expressed in activated T cells [159]. Upon activation, relative expression of the transcript with the longer 3′UTR decreased and protein expression significantly increased. This is consistent with a model in which use of alternative 3′UTRs prevents down-regulation by miRNAs, allowing up-regulation of protein production.

In general, longer 3′UTRs correlate with a relatively lower expression level, as indicated by experiments comparing the expression of isoforms differing only in their 3′UTR [159]. Notably, the average length of the 3′UTR in humans is more than twice as long as those of other mammals [140], which is indicative of an increase in regulatory elements in human genes. Although it is clear that miRNAs impact on 3′UTR length, other factors are also likely to contribute, potentially in a developmentally or tissue-specific manner. The relative position of motifs such as AREs within the 3′UTR can affect protein binding and regulation. The *β*_*2*_-adrenergic receptor (*β*_*2*_-AR) 3′UTR contains a number of AREs, although translational suppression seems to be primarily mediated by a 20nt ARE and a poly(U) region situated at the distal end of the 3′UTR. These motifs have been shown to bind T cell-restricted intercellular antigen-related protein (TIAR) that acts to repress translation, and HuR, an ARE-BP that can stabilise transcripts [80]. Recent experiments using reporter constructs demonstrated that the length of the 3′UTR is critical for these interactions, as TIAR binding was reduced in constructs with a shorter 3′UTR (~100 nt) in comparison to constructs with longer 3′UTRs (300 and 500 nt) [170]. HuR binding was not affected, indicating the two factors bind at non-overlapping sites and exert different roles on expression, increasing the complexity of regulation of this gene.

#### Secondary structure

Secondary structures within the 3′UTR are emerging as more important than previously envisioned. While the length of the 3′UTR is important, the secondary structure folding is also a vital determinant of translation efficiency, and mutations that change the secondary structure may result in disruption of expression. A study by Chen et al. [29] on 83 disease-associated variants in the 3′UTR of various human mRNAs found a correlation between the functionality of the variants and changes in the predicted secondary structure [29]. NMD is a quality control mechanism to remove mutated non-functional transcripts. Most commonly, the location of the nonsense mutation relative to the exon–exon junction complex determines the efficiency of NMD [24], but the 3′UTR may also play a role. The mechanisms of translation termination at premature termination codons (PTCs) have been shown to rely on the physical distance between the termination codon and the poly-A binding protein, PABPC1 [47]. This study found that extending the region between the normal termination codon and the poly-A tail resulted in NMD and that spatial rearrangements of the 3′UTR can modulate the NMD pathway [47].

Secondary structure of the 3′UTR is difficult to predict because of the multitude of factors binding the region, many of which are likely to induce structural changes. Factors can changes the spatial configuration of the region by disrupting mRNA folding, or by interacting with other factors resulting in the looping out of the mRNA in between [47]. The stem-loop RNA structure is the most common example of a secondary structure that can modify gene expression, and in the 3′UTR, this generally occurs through RNA-binding proteins. Brain-derived neurotrophic factor transcript (*BDNF*) contains an extended stem-loop structure that is responsible for the stability of the mRNA in neurons in response to Ca^+2^ signals [59]. The authors suggest that the stem-loop structure provides a scaffold for the interaction of a number of RNA binding proteins, non-coding RNAs and poly-adenylation factors in response to Ca^+2^. In *TNFα*, an ARE in the 3′UTR adopts a stem-loop structure that has been shown to modulate its affinity for various ARE-BPs [56]. These examples demonstrate that modulation of 3′UTR secondary structure by protein binding or other means can modulate *trans*-factor binding specificity and thus contributes to gene regulation at the post-transcriptional level.

#### Alternative 3′UTRs

Alternative poly-adenylation (APA) and alternative splicing are two mechanisms that can result in the production of mRNA isoforms differing in their 3′UTR. APA can occur because of the presence of multiple poly-adenylation sites, or by mutually exclusive terminal exons, and it is estimated that APA is utilised by ~50 % of human genes [38]. These mechanisms are very useful for complex organisms, as they provide a way in which transcripts can express the same protein but with varying expression levels and/or spatial localisation arising from variation in regulation from the 3′UTR [159]. Alternative 3′UTR use is an important aspect of developmentally- and tissue-specific gene expression [73, 77, 78, 186] (for an example, see [192] and large-scale changes in APA patterns have been associated with a number of different cancers [58, 113]. APA also plays an important role in isoform localisation [3]. The *HuR* gene is an ARE-BP that is involved in the stabilisation of many ARE-containing mRNAs. APA produces a number of *HuR* variants that differ in expression levels, and while the predominant transcript lacks AREs, a rare variant has been identified that contains functional AREs in the 3′UTR [1]. These AREs were found to bind HuR, thus inducing a self-up-regulation loop. Use of alternative 3′UTRs allows versatility of expression from a single gene.

### Conclusions

The 3′UTR is a versatile region that is enriched for regulatory elements and is vital for correct spatial and temporal gene expression. The 3′UTR is also emerging as a major hotspot for interactions with non-coding RNAs, with recent studies showing that large number of 3′UTRs are also expressed independently from the primary gene transcript and are likely to function in *trans* as non-coding RNAs of various lengths [122]. Further investigation into the regulatory functions of 3′UTRs has the potential to reveal even more complex pathways and interactions.

## Non-coding RNAs

Over the past decade, a wealth of evidence has revealed the pervasiveness and complexity of transcription throughout the human genome, with the majority of bases associated with at least one primary transcript [14]. As <1.5 % of the human genome codes for protein, this process results in widespread production of non-coding RNAs, of which there are many different types (interested readers are referred to reviews for each category), including miRNAs [76, 157, 190, 197], promoter-associated RNAs [55, 148], short interfering RNAs [132, 187], piwi-interacting RNAs [84, 96], small nuclear RNAs [39], natural antisense transcripts [53, 169] and long non-coding RNAs [31, 121, 145, 191], RNAs as extracellular signalling molecules [40], and long intronic non-coding RNAs [99]. Non-coding RNAs can be sense or antisense in orientation, transcribed in either direction and can originate from intergenic and intronic regions. Although there are some examples of non-coding RNAs conserved between distant species [185], the majority of non-coding RNAs seem to be species-specific, at least at the sequence level [70]. However, recent studies have shown that thousands of sequences within the mammalian genome possess conserved RNA secondary structures, while lacking any significant sequence conservation [177, 178]. Some non-coding RNAs are likely to function primarily through their secondary structures, which would result in relaxed sequence constraints and an underestimation of conservation between species. In any case, it is apparent that contrary to previous assumptions, a lack of conservation is not necessarily indicative of a non-functional sequence, and genome-wide evidence indicates that a significant proportion of non-coding RNAs perform functional roles [121].

Non-coding RNAs are key regulators of gene expression, acting at the individual gene level, regulating *cis* and *trans* interactions and contributing to control of transcription and translation, and on a genome-wide scale, regulating accessibility of chromatin and controlling gene pathways. Non-coding RNAs associate with each of the untranslated gene regions discussed in this review, contributing to the fine control of gene expression and increasing the complexity of the regulatory system. Transcribed regions including the 5′ and 3′UTRs, and intronic regions are also likely origins of non-coding RNA, following splicing and translation of the associated gene [122]. The use of RNA as a regulatory element has advantages because it can rapidly be synthesised and degraded [41], has structural plasticity and can modulate gene expression in response to external factors [4], and can act combinatorially to control complex interactions and regulatory pathways [106]. The discovery of non-coding RNAs, which were previously largely unnoticed, has come about due to advances in detection methods and technologies. Non-coding RNAs have now been identified spanning much of the genome, although they seem to be concentrated around gene promoters, enhancers and 3′UTRs [71]. This is indicative of a key role in the control of translation and stability. An in vitro study examining five different human cell types showed that the distribution of non-coding RNAs was non-random across the genome, differed among cell types, and that the distribution of sense and antisense transcripts were distinct [71]. In particular, antisense transcripts were concentrated around gene promoters and 3′UTRs, while sense transcripts were more prevalent around exons. Non-coding RNAs have now been found to control all aspects of gene expression.

A pseudogene is an imperfect copy of a functional gene, thought to arise during evolution by retrotransposition or duplication. Previously dismissed as non-functional DNA, evidence shows that some pseudogenes are fully transcribed, resulting in the production of natural antisense transcripts (NAT). NATs are involved in numerous vital cellular processes, including regulation of translation and stability, RNA export, alternative splicing, genomic imprinting, X inactivation, DNA methylation and modification of histones, and have also been shown to play roles in stress response and developmental processes [169]. NATs transcribed from pseudogenes have the potential to regulate sense transcripts arising from the functional parental gene through complementary binding, which has been shown in some cases to induce cleavage of the sense transcript [191]. Studies have shown that pseudogenes can also regulate their parental gene by interacting with enhancers, and that pseudogene transcripts can act as decoys for miRNAs that target the parental gene [143] (reviewed in [129]. It is estimated that up to 20 % of human pseudogenes are fully transcribed [199]. However, it is likely that pseudogenes also produce smaller non-coding RNAs that may regulate gene expression in *cis* or in *trans*. Transcription of pseudogenes often occurs in a tissue-specific manner, and the discovery that pseudogenes are capable of regulating tumour suppressors and oncogenes, and are often deregulated during cancer progression, indicates that they are important components of the non-coding RNA regulatory system (reviewed in [142]. The discovery that pseudogenes may function in the form of non-coding RNAs shows that previous assumptions about “non-functional” regions of the human genome should be challenged in the course of further research into non-coding RNAs.

Non-coding capacity is increased in primates in comparison to other animals. A comparison of pseudogenes across 28 vertebrate genomes showed that ~80 % of processed pseudogenes is primate specific, indicating that the rate of retrotransposition is increased in primates [199]. Non-coding capacity is especially increased in the brain, with non-coding RNA a major contributor to evolution of gene expression pathways [6]. RNA editing, a process by which bases are modified post-transcriptionally, is also predominantly active in the brain and is enriched in humans [111], increasing diversity of the transcriptome [138]. RNA editing is important as it allows adaptation to environmental stressors and may provide the basis for long-term memory and evolution of cognition throughout an individual’s lifetime [111]. RNA editing also occurs extensively in non-coding RNAs, again highlighting the importance of these transcripts in the brain. A comparative genomics study that looked at differences in humans that are highly conserved among other vertebrates identified 202 elements of significance, mostly in non-coding regions [144]. It is clear that non-coding RNAs are key players in regulation and genome control and increasing organism complexity.

In the past decade, research on non-coding RNAs has rapidly progressed, with hundreds of publications covering all known aspects of non-coding RNA function and regulation. For further information, readers are referred to reviews on various subtopics: intron evolution and function [103]; the significance of non-coding RNAs in organism complexity and evolution [104, 105, 108, 147]; functions of non-coding RNAs [2, 112], including regulation of transcription [70, 127], epigenetic processes [109, 127], structural roles [191], and response to environmental stimuli [180]; small regulatory RNAs in mammals [110]; non-coding RNAs in the human brain and development [107, 117] and in the nervous system [117]; and the involvement of non-coding RNAs in disease [173].

## Competing endogenous RNAs

Competing endogenous RNA (ceRNA) is a newly discovered mechanism by which RNA molecules can regulate expression of one another by competing for miRNAs. As mentioned previously, transcripts originating from pseudogenes have been found to regulate the expression of the corresponding gene [143]. Salmena and colleages proposed that this idea is not limited to pseudogene transcripts, but that all types of RNA transcripts can communicate with one another via matching miRNA response elements (MREs) [158]. This mechanism of communication between mRNAs adds a new level of complexity in which the expression of miRNAs is affected by the targets as well as vice versa, creating elaborate regulatory networks. The more shared MREs between mRNAs, the greater chance of communication and co-regulation [158]. ceRNA activity is influenced by the relative concentrations of the ceRNAs and their miRNAs in a given cell at a particular time, and also the binding capacity of the MREs.

The most well-studied example of ceRNA regulation involves the PTEN tumour suppressor gene. The PTEN-associated pseudogene has been shown to act as a ceRNA to regulate PTEN, with multiple conserved MREs allowing effective cross-talk between the two transcripts [143]. This was experimentally demonstrated by overexpression of the pseudogene 3′UTR that resulted in a significant increase in the levels of PTEN. Pseudogene transcripts are particularly suited as competing RNAs with the associated gene, because the high-sequence conservation implies that they contain the same MREs. In addition, a number of other protein-coding transcripts that regulate PTEN in a miRNA-dependent manner have been identified, such as SERINC1, VAPA and CNOT6L [176]. Studying ceRNA pathways is likely to be a useful tool for gaining insight into the changes that come about during tumour growth. Research using an in vivo mouse model of melanoma confirmed the ceRNA relationships discovered by Tay and colleagues [176] and validated the contribution of the ceRNAs in tumour growth and development [82].

Although mRNAs from protein-coding genes can act as ceRNAs, it has been suggested that non-coding RNAs are likely to be overrepresented as highly effective regulators as they may be specifically synthesised for the purpose of regulation and there is no interference from active translation [158]. A recent study identified a muscle-specific long non-coding RNA, linc-MD1, that plays an important role in muscle differentiation by acting as a ceRNA in mouse and human myoblasts [22]. It was found that linc-MD1 acts as a decoy for a number of miRNAs prevalent in muscle that are known to regulate the expression of multiple mRNAs. Targets of particular interest were MAML1 and MEF2C that are muscle-specific transcription factors involved in myogenesis. Data demonstrated that linc-MD1 communicates with these transcription factors as a ceRNA to regulate their expression [22]. Interestingly, the levels of linc-MD1 were found to be significantly reduced in Duchenne muscular dystrophy cells along with the delayed accumulation of muscle-specific markers MYOG and MHC, and it is possible that the disruption of this ceRNA pathway contributes to Duchenne muscular dystrophy pathology. The study also found that the activation of the linc-MD1 promoter correlates with the formation of a DNA loop at the beginning of myogenesis [22]. This is an example of how a ceRNA pathway can be activated when required and provide specific and sensitive control of mRNA levels in the cell.

ceRNA reveals a potential non-coding function of mRNAs that is separate to the protein function adding yet another layer of complexity to the genome. It also has implications for research in which a specific transcript is targeted for knockout or upregulation, as this would disrupt any ceRNA pathways involving that mRNA.

## Conclusion

The non-coding regions of the genome, including the 5′ and 3′UTRs, introns and intergenic regions, are vital for the precise regulation of gene expression and have evidently expanded during the evolution of complex organisms. In addition, the recently discovered ceRNA pathway also implicates a non-coding function for protein coding mRNAs, and evidence of pervasive transcription throughout the genome suggests that RNA is the most prevalent and versatile component of the gene regulatory network. This aim of this review was to discuss all the different mechanisms by which non-coding DNA and RNA contribute to the local and global expression profiles, with the numerous mechanisms of control outlined here demonstrating that this regulatory system is highly complex and sensitive. Adding to this complexity, regulation often occurs in a tissue- and developmental-specific manner, exponentially increasing the variation of expression from the genome. A typical gene is mostly non-coding sequence, and accumulated evidence shows that these regions facilitate specific expression of gene isoforms, in specific quantities, and enable rapid response to changing conditions.

The clear correlation between the relative amount of non-coding sequence and the complexity of an organism demonstrates that it is the control networks that are the most important for evolution. This is logical when one considers the enormous variation that can be produced from a single gene by layers of regulatory components acting in combinatorially to modulate gene expression. Complexity is increased by alternative mechanisms ways of gene processing, rather than the addition of more genes, as this allows an exponential increase in gene products rather than a linear increase. Humans have over 400 different cell types, including 145 types of neurons [183], all of which share the same DNA (with the exception of mature red blood cells and gametes). The differentiation of cell types has thus occurred through variation in the regulation of genes at all levels, from turning genes on or off, to subtle regulation arising from variation in non-coding RNA interactions. That the most significant changes in primates and humans in comparison to other organisms are found in the non-coding regions [83, 144] and the brain [6] is not surprising. A study looking at the nature of deletions of sequences in humans, that are otherwise highly conserved between chimpanzee and other mammals, found that the human-specific deletions fell almost exclusively in the non-coding regions, and were enriched near genes involved in neural function and steroid hormone signalling [116].

Non-coding RNAs are emerging as the most important, under-researched area of gene regulation and organism evolution. In order to appreciate and understand the complexity of regulation in the genome, it will be essential to utilise new technologies to detect and characterise non-coding RNAs, investigate how these interact with other elements, and elucidate their function. An understanding of the factors and elements involved in the regulation of a particular gene is of paramount importance when designing molecular therapies or when attempting to modulate the expression of a gene.
